# Ameliorative Effects of Gallic Acid on Cisplatin-Induced Nephrotoxicity in Rat Variations of Biochemistry, Histopathology, and Gene Expression

**DOI:** 10.1155/2021/2195238

**Published:** 2021-10-26

**Authors:** Zahra Eslamifar, Abbas Moridnia, Susan Sabbagh, Reza Ghaffaripour, Leila Jafaripour, Mahin Behzadifard

**Affiliations:** ^1^Department of Medical Laboratory Sciences, School of Paramedical Sciences, Dezful University of Medical Sciences, Dezful, Iran; ^2^Department of Immunology, School of Medicine, Dezful University of Medical Sciences, Dezful, Iran; ^3^Department of Anatomy, Faculty of Medicine, Dezful University of Medical Sciences, Dezful, Iran

## Abstract

**Background:**

Cisplatin is a powerful chemotherapeutic drug mainly used in the treatment of solid tumors. Aggregation of the drug in renal proximal tubule cells causes nephrotoxicity and renal failure. Investigations showed nephrotoxicity as Cisplatin's dose-limiting side effect. One of the Cisplatin toxicity mechanisms is generation of reactive oxygen species, which leads to oxidative stress and renal damage. The purpose of this study was evaluation of the modulating effects of Gallic acid on Cisplatin-induced variations including Caspase-3 and Clusterin expression and histopathological and biochemical parameters in adult male Wistar rats.

**Method:**

Rats were kept under standard condition of temperature, light, and humidity. The animals were divided into 4 groups: GpI: control group (received distilled water for 10 days); GpII: Gallic acid (alone) (50 mg/kg bw, once a day for 10 days); GpIII: Cisplatin (alone), single dose (6 mg/kg bw, I.P. on 5th day of study); GpIV: Gallic acid (50 mg/kg bw, once a day for 10 days) and also injected with single dose of Cisplatin (6 mg/kg bw, I.P., on 5th day of study). After 10 days, all rats were anaesthetized and plasma collected to estimate urea, creatinine, and uric acid. The right kidneys were removed for the study of gene expression and biochemical parameters. The left kidneys were used for histopathological studies.

**Results:**

The Cisplatin-induced nephrotoxicity was evident from the elevated levels of creatinine, urea, uric acid, and renal tissue MDA and also decreased levels of SOD, CAT, GPX, and GSH in renal tissue. Administration of Gallic acid significantly modulated nephrotoxicity markers, gene expression variations, and histopathological damage.

**Conclusion:**

Outcomes of the present investigation suggest that Gallic acid provides protection against CP-induced nephrotoxicity, but for application in people, further studies are needed.

## 1. Introduction

Cisplatin (cis-diamminedichloroplatinum-II) is a powerful chemotherapeutic drug predominantly used in the treatment of solid tumors [1].

The kidney in addition to playing its role as eliminator of endogenous and exogenous waste materials, including drugs, stores some of these substances in the proximal tubular section. In the treatment with Cisplatin (CP), renal tissue accumulates this drug to a higher level than other tissues and organs.

Cisplatin concentration in the epithelial cells of renal proximal tubules was detected to be about 5 times higher than serum [2]. Aggregation of the drug leads to strong toxicity in renal proximal tubule cells and finally causes tissue destruction, low perfusion, and renal failure [3, 4]. Low renal perfusion indicates nephrotoxicity and necrosis of the terminal portion of the proximal tubule and finally determines renal tissue fate [5].

Nearly 25–35% of subjects following a single dose of Cisplatin develop evidence of renal dysfunction [6]. Preclinical investigations showed nephrotoxicity and renal dysfunction as one of Cisplatin's dose-limiting side effects [5].

Acute renal failure is a considerable motivating force of mortality and morbidity development among Cisplatin chemotherapy recipient subjects. Nephrotoxicity is distinguished by tubular dilation, tubular cell vacuolization, loss of microvilli, and condensation of nuclear chromatin. Tubular destruction can be the loss of only the margin of the epithelial cell brush to tubular necrosis in severe renal damage. Diminished glomerular filtration and enhancement of blood urea nitrogen and plasma creatinine represent Cisplatin-induced renal failure [6–9].

Multifactorial mechanisms are involved in Cisplatin-induced nephrotoxicity including the production of free radicals such as superoxide and hydroxyl radicals, mitochondrial dysfunction, and increased activity of calcium independent nitric oxide synthase and ultimately apoptosis. One of the mechanisms and processes that have been detected to play Cisplatin toxicity is its impact on DNA synthesis and repairs, which causes cell cycle inhibition [9, 10].

Mitochondrial irregularity is a key event in Cisplatin-induced acute renal failure. Cisplatin collection in the mitochondria causes the generation of reactive oxygen species leading to oxidative stress resulting in nephrotoxicity and renal damage [11, 12].

Investigations have also presented that Cisplatin induces the tumor-suppressor protein p53. This affects apoptosis via the receptor-tumor necrosis factor interaction, and Caspases that cause mitochondrial disorder also affect the calcium signaling by stress on the endoplasmic reticulum [9, 13, 14].

Despite the side effects of Cisplatin, it is the preferred treatment in chemotherapy due to its high efficacy and low cost [15].

There are several anti-inflammatory and antioxidant factors that have ameliorative effects on the potential side effects of Cisplatin [16–18].

3,4,5-Trihydroxybenzoic acid called Gallic acid (GA) is an antioxidant product, present in some natural ingredients including green tea, red wine, pineapples, strawberries, lemons, bananas, sumac, gallnuts, tea leaves, witch hazel, apple peels, and oak bark [19–21].

Gallic acid, as a powerful chelating factor, protects tissues against oxidative stress due to antioxidant and anti-inflammatory effects [22–24]. This valuable compound does not only maintain the integrity of cell membrane but also increases the regenerative capacity of the kidney and liver [25]. Furthermore, Gallic acid and its derivations have anticancer properties due to antioxidant and anti-inflammatory features [26].

Clusterin gene expression is upregulated in response to numerous types of renal toxicity and diseases [27]. Wang et al. showed that Clusterin gene expression was highly induced in rats after treatment with Cisplatin [28].

Reactive oxygen species show a significant role in apoptosis by inducing the Caspase activity. Among the Caspase family, Caspase-3 specially is a main apoptotic effector leading to cytoskeletal decomposition, nuclear destruction, and other changes associated with apoptosis [29, 30].

Mechanisms related to inducing the Caspase-3 activity are all known to be involved in Cisplatin-induced tubular apoptosis. Caspase inhibition markedly reduces nephron injury in Cisplatin therapy [31].

The recognition of natural antioxidants and their possible function on side effects of chemotherapeutic drugs provides a valuable plan in the treatment of patients.

The main purpose of our study was to estimate the modulating effects of Gallic acid on Cisplatin-induced variations on Caspase-3 and Clusterin expression and nephrotoxicity in adult male Wistar rats.

## 2. Materials and Methods

Cisplatin and Gallic acid were purchased from Sigma Chemical Co (St Louis, MO, USA). The Gallic acid was dissolved in distilled water just before the experiments. Other materials used were of analytical grade.

### 2.1. Animals

Healthy adult male Wistar rats (12–14 weeks old; weighing 200 ± 20 g) were purchased from the Animal House of Ahvaz Jundishapur University of Medical Sciences, Ahvaz, Iran. The rats were housed in clean polypropylene cages with free access to standard rat chow and water under controlled conditions of humidity (65% ± 5), temperature (25 ± 2°C), and air ventilation with a 12 h light/dark cycle. The maintenance protocol of animals was approved by the Institutional Animal Ethics Committee (Registration number IR.DUMS.REC.1398.008; Proposal No. 1532: dated 06.02.2019), Dezful University of Medical Sciences, Dezful, Iran.

### 2.2. Experimental Design

32 male Wistar rats were divided into 4 groups including experimental groups and control group (8 rats in each group).

Group I received distilled water for 10 days as the control group (cnt group). Group II (gal group) was administered by gavage with Gallic acid alone (50 mg/kg bw, once a day for 10 days) starting from day 1 and continuing daily for 10 days.

Group III (cis group) was injected intraperitoneally with a single dose of Cisplatin alone (6 mg/kg bw, injection on 5th day).

Group IV (cis-gal group) was administered by gavage with Gallic acid (50 mg/kg bw, once a day for 10 days) and also injected with a single dose of Cisplatin (6 mg/kg bw, I.P., on 5th day of study).

All rats were anaesthetized with diethyl ether on the 10th day of investigation, and then, blood samples from the heart were collected in heparinized vials, and plasma was used to estimate of biochemical parameters (B. urea, P. creatinine, and P. uric acid using commercially available diagnostic kits).

The right kidneys were removed and frozen in -70°C until homogenization and the study of gene expression and biochemical parameters. The left kidneys were placed in 10% formalin for histopathological studies.

### 2.3. Investigations of Biochemical Parameters

10 times the phosphate-buffered saline (PBS) was added to the homogenized kidney. It was centrifugated at 4°C with 12000 rpm for 15 minutes. The supernatant was used to measure malondialdehyde (MDA), glutathione (GSH), glutathione peroxidase (GPX), superoxide dismutase (SOD), and catalase (CAT). In the present study, the contents of malondialdehyde (MDA) of renal tissue were evaluated according to the thiobarbituric acid (TBA) method, similar to the method described by Buege and Aust [32]. Absorption was read at 532 nm by spectrophotometer (Hitachi's U-2000 Double-Beam UV/Vis Spectrophotometer). Renal levels of GSH were determined in accordance with the Ellman method [33]. GPX activity was measured by the Rotruck et al. method. In brief, tert-butyl hydroperoxide and hydrogen peroxide (H_2_O_2_) were mixed with the kidney sample, and then, its absorption was read by the ELISA reader at 420 nm [34, 35]. SOD activity was measured by the Minami et al. method [36]. For the CAT reaction, 0.7 ml of potassium phosphate buffer (pH: 7.0), 0.1 ml of hydrogen peroxide (H_2_O_2_), and 100 *μ*l of a homogenized renal sample were mixed, and its absorption at 240 nm was read by a spectrophotometer (Hitachi's U-2000 Double-Beam UV/Vis Spectrophotometer). [37].

### 2.4. Histopathological Investigations

The rat's left renal tissue was removed, washed with ice-cold physiological saline solution, and fixed in 10% neutral buffered formalin solution for 24 h. Routine processing and paraffin embedding were done, and the tissue was sectioned at 4-5 *μ*m thickness by a rotary microtome. Sections were stained with hematoxylin-eosin (H&E) dyes and observed in a magnification of 100x under a light microscope (Olympus BX 52, Tokyo, Japan) [38].

For each kidney, composed of slides of control and experimental groups (at least 50 samples), the glomerulus diameter and thickness of proximal convoluted tubule (PCT) were measured by using an image analysis software; the Motic Images Plus 2.0 and the average of measured data were calculated.

Additionally, five stained sections of each renal tissue were assessed for these criteria: vacuolization of proximal cell, congestion of red blood cells, leukocyte infiltration, eosinophilic casts necrosis of PCT cells, and hemorrhage. Then, the percentage of acute renal tubular necrosis was classified and scored by the semiquantitative method, such as 0 point: normal; 1 point: <10%; 2 points: 10-25%; 3 points:26-75%; and 4 points: >75%. Finally, for each measure, the average percentage was determined [39].

### 2.5. RNA Extraction, cDNA Synthesis, and Real-Time PCR

The total RNA was extracted from rat kidney tissue using BioFACT™ Total RNA Prep Kit (Ver. 2.0, South Korea), according to the manufacturer's instruction. In the first step, liquid nitrogen was added to the samples and tissues were crushed separately. The samples were transferred to RNase-free tubes and stored on ice. Then, 1 ml of tissue lysis buffer was added to the tubes that were homogenated by an ultrasonic sonicator. Finally, the RNA purity was checked with a NanoDrop ND-1000 UV-Vis Spectrophotometer (Thermo Fisher Scientific, Waltham, MA, USA). In addition, complementary DNAs (cDNAs) were synthesized by the BioFact™ RT Series Kit (BioFact, South Korea) according to the manufacturer's instructions. The real-time qPCR was done for *CASP3* and *Clusterin* gene expression using the SYBR Green Master Mix Kit (BioFact, South Korea). The *GAPDH* gene was used for internal control and normalization. The primers were as follows: *Clusterin* F: 5′ TACAACGAGCTGCTTCATTC 3′, R: 5′ AGAATGGGTTGTCACTGTGG 3′; *CASP3* F: 5′ CTGGACTGTGGCATTGAGAC 3′, R: 5′ TCCAGGAATAGTAACCAGGTG 3′; and *GAPDH* F: 5′ CTCATGCGACTTCAACAGC 3′, R: 5′ CGTCTACATTGTCATACCAGG 3′. The ABI StepOnePlus (Applied Bio systems, Foster City, CA, USA) instrument and *ΔΔ*Ct method were used for data analysis of the qPCR experiment.

### 2.6. Statistical Analysis

Data were analyzed by the Shapiro-Wilk normality test for normality. Parameters that showed normal distribution were used as a one-way analysis of variance (ANOVA). In case of significance between groups, Tukey's multiple-comparison posttest was used, and the data are expressed as mean ± standard deviation (SD). Nevertheless, data that did not show a normal distribution used nonparametric tests such as Kruskal-Wallis tests, and the data were expressed as median (min–max). Statistical analysis was performed by GraphPad Prism 8.0. A statistical probability of *P* value < 0.05 was considered statistically significant.

## 3. Results

### 3.1. Ameliorative Effects of Gallic Acid on Plasma Kidney Parameters

The effects of Gallic acid (50 mg/kg bw, orally) on Cisplatin- (6 mg/kg bw, IP) induced nephrotoxicity were evaluated by the study of plasma biochemical parameters.

**Figure 1:**
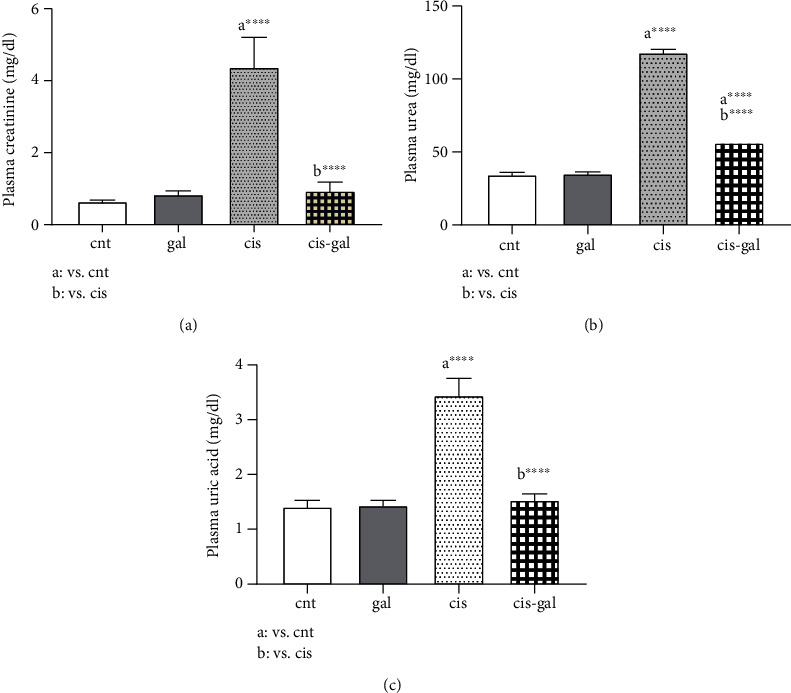
Effect of Gallic acid on Cisplatin-induced nephrotoxicity as measured by (a) plasma creatinine, (b) plasma urea, and (c) plasma uric acid. Nephrotoxicity in rats was induced by a single dose of Cisplatin (6 mg/kg bw, IP) and Gallic acid (50 mg/kg bw, gavage) administered 5 days prior to CP treatment and continued till the end of the experiment (5 days). ^∗∗∗∗^*P* < 0.0001; values are expressed as mean ± SD (*n* = 6). The results show that Gallic acid attenuated Cisplatin-induced renal toxicity.

The nephrotoxicity effect of Cisplatin was evident from the elevated levels of plasma renal markers including P. creatinine, B. urea, and P. uric acid observed in the cis group (Figure 1). It was demonstrated that glomerular filtration was damaged. Administration of Gallic acid prior to and following Cisplatin treatment significantly decreased nephrotoxicity (cis-gal group IV, compared to cis group III) (Figure 1, *P* < 0.0001).

### 3.2. Ameliorative Effects of Gallic Acid on Renal Tissue Lipid Peroxidation

**Figure 2:**
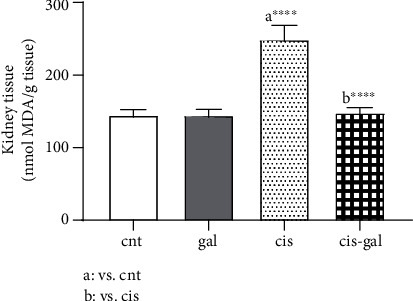
Effect of Gallic acid on Cisplatin-induced lipid peroxidation (kidney tissue MDA). Rats were injected with Cisplatin and Gallic acid as described in Figure 1. ^∗∗∗∗^*P* < 0.0001; values are expressed as mean ± SD (*n* = 6). The results show that Gallic acid treatment ameliorated the Cisplatin-induced increase in lipid peroxidation.

Lipid peroxidation was investigated in terms of thiobarbituric acid reactive substances (TBARS), expressed as the malondialdehyde (MDA) level. In this study, MDA were remarkably increased in the kidney tissue of the cis group compared to the cnt group (*P* < 0.0001). The elevated MDA level was ameliorated by treatment with Gallic acid (*P* < 0.0001) (cis group III compared to cis-gal group IV) (Figure 2). The administration of Gallic acid alone did not increase the level of MDA compared to the control rats. Indeed, the MDA concentrations were similar in the cnt group and gal group (*P* < 0.05). Figure 2 shows the results in the 4 groups.

### 3.3. Effects of Gallic Acid Treatment on Renal Tissue Antioxidant Parameters

For evaluating the role of Gallic acid in attenuation of Cisplatin-induced oxidative stress, the effect of Cisplatin and Gallic acid alone and in combination was investigated on enzymatic and nonenzymatic antioxidant defense parameters.

**Figure 3:**
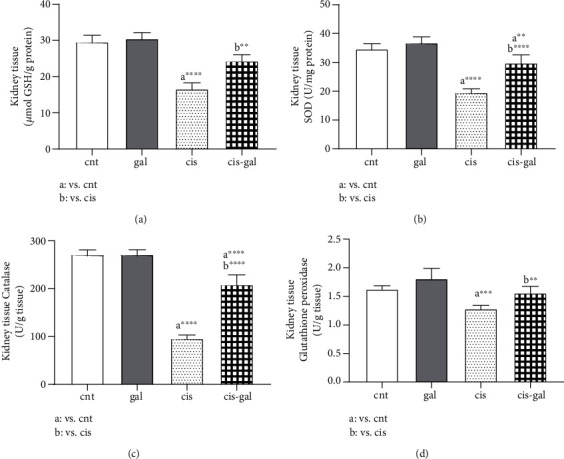
Effect of Gallic acid on Cisplatin-induced decrease in (a) GSH, (b) SOD, (c) CAT, and (d) GPX in kidney tissue. Rats were treated with Cisplatin and Gallic acid as described in Figure 1. ^∗∗^*P* < 0.01, ^∗∗∗^*P* < 0.001, and ^∗∗∗∗^*P* < 0.0001; values are expressed as mean ± SD (*n* = 6). The results show that Gallic acid treatment ameliorated the Cisplatin-induced decrease in SOD, catalase, GSH, and glutathione peroxidase.

The effect of treatment with Gallic acid on Cisplatin-induced alterations in the levels of kidney tissue GSH and enzymatic antioxidant parameters including SOD, CAT, and GPX are shown in Figure 3.

Compared to the control, the rats injected with Cisplatin were shown to be remarkably reduced in renal tissue GSH (*P* < 0.0001). Treatment of animals with Gallic acid ameliorated the Cisplatin-mediated decrease in GSH (cis group vs. cis-gal group, *P* < 0.01).

Gallic acid did not induce a statistically significant increase in kidney tissue glutathione compared to control (*P* < 0.05).

The levels of antioxidant enzyme activity were also reduced significantly in Cisplatin-treated animals compared to control (SOD *P* < 0.0001, CAT *P* < 0.0001, and glutathione peroxidase *P* < 0.001) and also increased in rats treated with Gallic acid plus Cisplatin compared to the cis group (SOD *P* < 0.01, CAT *P* < 0.0001, and glutathione peroxidase *P* < 0.01).

Therefore, it was observed that oral administration of Gallic acid prior to and following Cisplatin treatment considerably ameliorated Cisplatin-induced decrease in the activities of the antioxidant enzyme.

### 3.4. Effects of Gallic Acid Treatment on Renal Tissue Histopathological Changes

**Figure 4:**
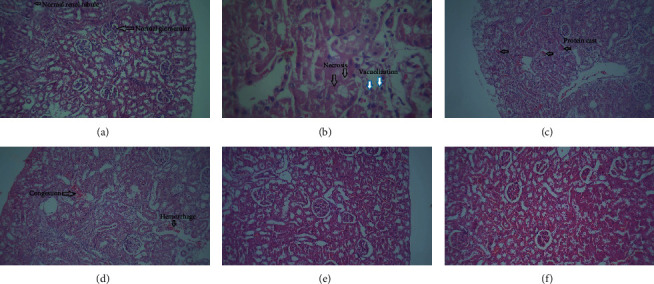
H&E staining of renal tissues, magnification ×100. (a) Control group, light microscopy examination of renal tissues in control group showed normal structure of glomerulus, renal cortex, and interstitium with no evidence of acute cell necrosis. Photomicrograph of a renal section taken from Gallic acid-supplemented rats had almost normal structure of glomerulus, renal cortex, and interstitium without acute necrosis (b–d). But photomicrograph of a renal section taken from cisplatin-treated rats showing marked vacuolization, necrosis of tubular cells, protein cast, and congestion of vessels (e). Photomicrograph of a renal section taken from Gallic acid-supplemented cisplatin-treated rats showing that treatment with Gallic acid remarkably attenuated renal tissue injury (f).

The histopathological changes of administration of Cisplatin and Gallic acid in kidney tissues are shown in Figure 4.

One-way ANOVA did not show a significant difference between the groups in terms of the diameter of glomeruli (*F* (3, 17) = 2.73, *P* = 0.076) (Table 1). One-way ANOVA showed a significant difference between the groups in terms of the thickness of proximal convoluted tubules (*F* (3, 15) = 6.81, *P* = 0.004). In this study, thickness of proximal convoluted tubules was significantly reduced in the Cisplatin group compared to the control group (*P* = 0.002). Nevertheless, no significant difference was observed in the Cisplatin group vs. the Cisplatin-Gallic group (*P* = 0.077) and the control group vs. the Gallic group (*P* = 0.124) (Table 1).

A Kruskal–Wallis test showed a significant difference among the groups in terms of the cell necrosis in the kidney (*P* = 0.002). Cell necrosis was significantly increased in the Cisplatin group compared to the Control group (*P* = 0.006). Cell necrosis was significantly decreased in the Cisplatin-Gallic group compared to the Cisplatin group (*P* = 0.001) (Table 1).

In the present study, the Kruskal–Wallis test showed a significant difference among the groups in the eosinophilic casts in the kidney (*P* < 0.0001). In the study, eosinophilic casts have a significant difference in the control group compared with the Cisplatin group (*P* = 0.006) and the Cisplatin group compared with Cisplatin-Gallic group (*P* = 0.001) (Table 1).

In this study, analyzing the vacuolization using a Kruskal–Wallis test revealed a significant difference among the groups (*P* = 0.003). Vacuolization was significantly increased in the Cisplatin group compared to the Control group (*P* = 0.006). Vacuolization was significantly decreased in the Cisplatin-Gallic group compared to the Cisplatin group (*P* = 0.008) (Table 1).

In addition, a Kruskal–Wallis test showed a significant difference in the congestion (*P* = 0.014), hemorrhage (*P* < 0.0001), and leukocyte infiltration (*P* < 0.0001). However, no significant difference was observed in the control group vs. Cisplatin group and the Cisplatin group vs. Cisplatin-Gallic group (Table 1).

### 3.5. Effects of Gallic Acid on Expression Level of CASP3 and Clusterin Genes in Rat Kidney Tissue

**Figure 5:**
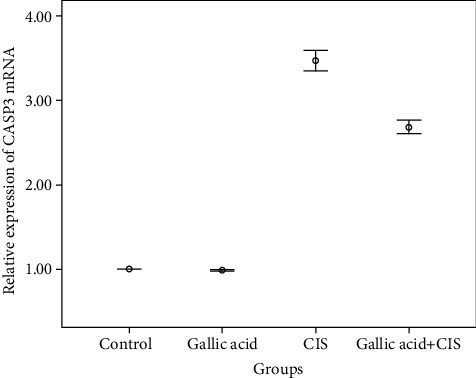
Effect of Gallic acid on Cisplatin-induced increase in expression of *CASP3* in kidney tissue. Rats were treated with Cisplatin and Gallic acid as described in Figure 1. The results show that Gallic acid treatment improved the Cisplatin-induced increase in gene expression.

The expression levels of *CASP3* and Clusterin genes were assessed by RT-qPCR in the control (cnt), Gallic acid (gal), Cisplatin (cis), and Cisplatin+Gallic acid (cis-gal) groups after ten days of treatment. The cis-gal group showed significant downregulation in *CASP3* mRNA expression in rat kidney tissue compared to the cis group alone (*P* < 0.05) (Figure 5). The expression level of the *Clusterin* gene was not detectable in all groups.

## 4. Discussion

Cisplatin is still an effective and practical anticancer agent. However, in 30% of patients, it is clinically associated with acute kidney injury [9, 40].

This antitumor drug inhibits the mitochondrial respiratory complex in renal tubular cells, resulting in generation of reactive oxygen species (ROS) and finally tissue and organ damage [41].

The ROS generation and oxidative stress in renal tissue have resulted in lipid peroxidation and changes in enzymatic and nonenzymatic antioxidant system and the gene expression [9, 42, 43].

Irregularities in the structure and function of renal tissue are believed to be associated with inflammation and oxidative stress production. Inflammation was caused through chemokines such as tumor necrosis factor [9]. Some studies report that apoptosis is involved in CP-induced nephrotoxicity [44–46].

Some antioxidant compounds prevented CP-induced toxicity such as melatonin [47], selenium [48], vitamin E and vitamin C [43, 49, 50], some plants [51], modulators of nitric oxide, agents interfering with Cisplatin metabolic pathways, and antiapoptotic and cytoprotective agents [16, 52–54].

Several phenolic acid compounds, such as aqueous extract of Dendrobium nobile Lindl, protocatechuic acid, and ferulic acid, have shown conservation against nephrotoxicity and hepatotoxicity induced by Cisplatin [55–57].

Gallic acid as an antioxidant is a phenolic acid compound that can scavenge peroxyl radicals and oxidative damage in tissues [58, 59]. Numerous investigations have shown that Gallic acid has considerable antioxidant activity [60] and exhibits anti-inflammatory feature in oxidative conditions that can cause tissue damage [61, 62].

Gallic acid prevents methotrexate-induced nephrotoxicity [23] and sodium arsenite-induced toxicity in the rat's liver and kidney [63].

In renal tissue, Cisplatin is converted into more reactive molecules. Therefore, the production of ROS, oxidative stress, accumulation of MDA, and depletion of antioxidant enzyme activity occur. GSH is a significant ROS scavenger and is required to maintain cell safety. CAT and GPX decompose H_2_O_2_, and the dismutation of the superoxide anion is done by SOD. SOD, CAT, and GPX are antioxidant enzymes that are necessary to improve kidney function [64].

Disorder in the kidney function by Cisplatin is demonstrated via the attenuation of renal tissue enzyme function such as SOD, CAT, and GPX and the increase of B. urea, P. uric acid, and P. creatinine and also MDA generation [30, 65–67].

In this study, nephrotoxicity was induced by injecting a single I.P. dose of Cisplatin (6 mg/kg bw) [68]. Renal damage was confirmed by variation of the gene expression of Caspase-3 and histological changes along with increased nephrotoxicity markers including P. creatinine, P. uric acid, B. urea, and lipid peroxidation marker (MDA).

We indicated that the use of Gallic acid significantly improved renal function with an ameliorating effect on biochemistry and histopathological marker and the expression of Caspase-3 in Cisplatin-induced nephrotoxicity.

This further confirms the antioxidative feature of Gallic acid in Cisplatin nephrotoxicity.

The anti-inflammatory and antioxidant feature of Gallic acid may play a key role of modulatory effect in Cisplatin-induced toxicity [62, 69, 70].

Apoptosis due to reperfusion ischemia and treatment with melatonin by prevention of Caspase-3 activity has also been reported [71].

Also, it was reported that C-phycocyanin significantly inhibited the doxorubicin-induced ROS generation and apoptosis by decreasing Caspase-3 activity [72].

The present study showed that the expression of Caspase-3 was downregulated in the cis-gal group that was confirmed by qRT-PCR. Therefore, the protective effect of orally prescribed Gallic acid, as a natural antioxidant having free radical scavenging effects, was demonstrated by the prevention of Caspase-3 activity.

These preclinical findings present Gallic acid to light as a nephroprotective agent against Cisplatin in male Wistar rats.

## 5. Conclusion

The common method for cancer treatment is chemotherapy with Cisplatin, but this drug has side effects such as nephrotoxicity.

Our study showed the modulatory effect of Gallic acid as an antioxidant in rat kidney tissue. Accordingly, Gallic acid can be an excellent treatment scope in reducing the oxidative stress and finally severity of Cisplatin-induced nephrotoxicity.

These studies are important since Cisplatin can affect the reabsorptive functions of the renal tissue leading to acute kidney injury.

Although the outcomes of the present investigation suggest that Gallic acid provides protection against CP-induced renal toxicity by the prevention of oxidative stress, alternative possible mechanisms cannot be ruled out.

In conclusion, Gallic acid markedly reduces Cisplatin-induced nephrotoxicity, but further studies are necessary to exclude the fact that Gallic acid may affect the therapeutic efficacy of Cisplatin in people. In the absence of such evidence, despite the marked renal protection, definitive evaluation of the potential therapeutic importance of Gallic acid in people as an adjuvant in chemotherapy cannot be practical.

## Figures and Tables

**Table 1:** Morphologic and histopathological renal parameters of the control group and experimental groups. Diameter of glomeruli and thickness of proximal convoluted tubules were analyzed by one-way ANOVA and expressed by mean ± SD. Other parameters were analyzed with Kruskal-Wallis test and expressed by median (min–max).

Groups	Diameter of glomeruli, *μ*m (mean ± SD)	Thickness of proximal convoluted tubules, *μ*m (mean ± SD)	Cell necrosis, median (min, max)	Eosinophilic casts, median (min, max)	Vacuolization, median (min, max)	Congestion, median (min, max)	Hemorrhage, median (min, max)	Leukocyte infiltration, median (min, max)
Control	(144/9 ± 9/464)	(20/60 ± 6/330)	0.50 (0.00, 1.00)	0.00 (0.00, 0.00)	0.00 (0.00, 0.00)	0.50 (0.00, 1.00)	0.00 (0.00, 1.00)	0.00 (0.00, 0.00)
Gallic acid	(135/1 ± 5/494)	(15/50 ± 0/4082)	0.50 (0.00, 1.00)	0.00 (0.00, 0.00)	0.00 (0.00, 1.00)	0.00 (0.00, 0.00)	0.00 (0.00, 0.00)	0.00 (0.00, 1.00)
Cisplatin	(128/1 ± 10/20)	(11/47 ± 1/730)	3.00 (3.00, 3.00)	1.00 (1.00, 2.00)	1.00 (1.00, 2.00)	1.00 (1.00, 3.00)	1.00 (0.00, 1.00)	1.00 (0.00, 1.00)
Gallic acid+Cisplatin	(138/5 ± 11/57)	(16/34 ± 1/058)	1.00 (0.00, 2.00)	0.00 (0.00, 0.00)	0.00 (0.00, 1.00)	0.50 (0.00, 1.00)	0.00 (0.00, 1.00)	0.00 (0.00, 1.00)
*P* value	Cnt vs. cis (0.0624) Cis vs. cis-gal (0.2633) Cnt vs. gal (0.5135)	Cnt vs. cis (0.0022) Cis vs. cis-gal (0.077) Cnt vs. gal (0.1246)	Cnt vs. cis (0.006) Cis vs. cis-gal (0.001) Cnt vs. gal (1.000)	Cnt vs. cis (0.006) Cis vs. cis-gal (0.001) Cnt vs. gal (1.000)	Cnt vs. cis (0.006) Cis vs. cis-gal (0.008) Cnt vs. gal (0.686)	Cnt vs. cis (0.164) Cis vs. cis-gal (0.101) Cnt vs. gal (0.343)	Cnt vs. cis (0.412) Cis vs. cis-gal (0.234) Cnt vs. gal (0.686)	Cnt vs. cis (*P* = 0.164) Cis vs. cis-gal (0.234) Cnt vs. gal (0.686)

## Data Availability

Upon reasonable request, the data supporting the results of this article will be made available by the corresponding responsible author.
